# Velocidade de Onda de Pulso de 8,2m/s como Limiar Associado à Presença de Lesão de Órgão Alvo Cardiovascular

**DOI:** 10.36660/abc.20220934

**Published:** 2023-10-26

**Authors:** Sayuri Inuzuka, Priscila Valverde de Oliveria Vitorino, Adriana Sebba Barroso, Fabrício Galdino Magalhães, Andrea Cristina Sousa, Robson Pierre Pacífico Alves, Victoria Alves Melo, Luiz Fernando de Oliveira, Ana Luiza Lima Sousa, Paulo Cesar B. Veiga Jardim, Antonio Coca, Weimar Kunz Sebba Barroso

**Affiliations:** 1 Universidade Federal de Goiás Goiânia GO Brasil Universidade Federal de Goiás - Liga de Hipertensão Arterial, Goiânia, GO – Brasil; 2 Pontifícia Universidade Católica de Goiás Escola de Ciências Sociais e da Saúde Goiânia GO Brasil Pontifícia Universidade Católica de Goiás - Escola de Ciências Sociais e da Saúde, Goiânia, GO – Brasil; 3 Hospital Clinic University of Barcelona Barcelona Espanha Hypertension and Vascular Risk Unit. Hospital Clinic. University of Barcelona, Barcelona – Espanha

**Keywords:** Rigidez Vascular, Análise de Onda de Pulso, Pressão Arterial, Hipertensão

## Abstract

**Fundamento:**

Estudos prévios estabeleceram valores de normalidade e de referência da Velocidade de Onda de Pulso (VOP). Porém, qual valor de VOP que apresenta a associação mais forte com biomarcadores cardiovasculares ainda é pouco conhecido.

**Objetivo:**

Identificar o valor de VOP com maior possibilidade de estar associado com hipertrofia ventricular esquerda (HVE), aumento da espessura íntima-média carotídea (EIMC), e presença de placas carotídeas em pacientes hipertensos.

**Métodos:**

Este é um estudo transversal de 119 pacientes. Análise de curvas características de operação do receptor (ROC) foi realizada para cada biomarcador cardiovascular. A diferença estatística foi estabelecida em p<0,05.

**Resultados:**

Segundo análises das curvas ROC, valores de VOP de 8,1m/s para HVE, 8,2m/s para EMIC aumentada e 8,7m/s para a presença de placa carotídea foram encontrados, respectivamente. O valor de VOP de 8,2m/s foi definido como melhor o parâmetro para encontrar os três biomarcadores de LOA. A VOP acima de 8,2m/s associou-se ao aumento da EMIC (p = 0,004), à presença de placas carotídeas (p = 0,003) e à HVE (p < 0,001). A VOP acima de 8,2m/s apresentou maior sensibilidade para EMIC aumentada (AUC = 0,678, sensibilidade 62,2), HVE (AUC = 0,717, sensibilidade 87,2), e presença de placas (AUC = 0,649, sensibilidade 74,51) na análise das curvas ROC.

**Conclusão:**

O valor de 8,2m/s de VOP foi mais sensível em identificar, precocemente, a existência de biomarcadores cardiovasculares de LOA.

## Figura Central

**Figure:**
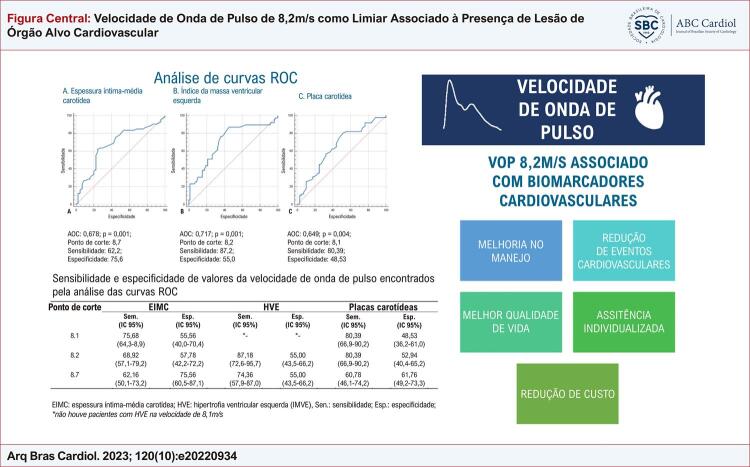
: Velocidade de Onda de Pulso de 8,2m/s como Limiar Associado à Presença de Lesão de Órgão Alvo Cardiovascular

## Introdução

Muitos pacientes hipertensos apresentam lesões subclínicas em estágios iniciais da doença, que não são identificadas por modelos tradicionais de avaliação.^1 - 3^ Segundo as principais diretrizes de hipertensão, testes complementares mais específicos para análise de biomarcadores são usados para a identificação precoce de dano cardiovascular (CV).^4 - 6^

A velocidade de onda de pulso (VOP) carotídeo-femoral é o padrão-ouro para medida da rigidez arterial, por ser um método invasivo, simples, preciso, reprodutível, e ter valor preditivo.^7 , 8^ Valores estratificados da VOP estão disponíveis para indivíduos sadios e aqueles com risco CV aumentado. Ainda, foi estabelecida uma associação entre VOP e lesão de órgão alvo (LOA) em pacientes com hipertensão.^9 - 11^

Estudos mostraram que a VOP é um preditor de eventos e mortalidade CV.^3 , 12 - 14^ O uso da VOP, além de fatores de risco CV tradicionais, melhora a estratificação de risco.^15 , 16^ Um ponto de corte de 10m/s para a VOP foi estabelecida como uma definição de LAO vascular em estudos prévios e diretrizes.^4 - 6^ Além disso, uma VOP acima de 10m/s foi associada a biomarcadores de mudanças estruturais na câmara do ventrículo esquerdo e artérias carótidas, bem como a um aumento na mortalidade CV.^16 - 18^

A rigidez arterial associada com idade e sexo, e valores estratificados usando o método tonométrico foram estabelecidos em estudos prévios, a maioria deles conduzido na Europa.^9 , 19 , 20^ Um estudo europeu^20^ distinguiu valores “normais” de “valores de referência”, Enquanto valores “normais” fornecem uma faixa fisiológica, os valores “de referência” indicam até quais valores uma população não apresenta doenças CVs observáveis. Recentemente, valores de referência usando o método oscilométrico^10 , 11 , 21^ foram classificados em dois grupos, com e sem fatores de risco CVs, e estratificados por idade e sexo.^11^

Existe uma lacuna na literatura quanto à determinação de um percentil ideal para VOP para identificar um valor normal ou o início de dano CV. O presente estudo teve como objetivo identificar o valor da VOP, não como uma LOA, mas aquele com associação mais forte com os seguintes biomarcadores: espessura íntima-média carotídea (EIMC), presença de placas ateromatosas, e hipertrofia ventricular esquerda (HVE).

## Métodos

Este estudo transversal foi conduzido entre outubro de 2018 e março de 2019 em um serviço ambulatorial multidisciplinar de um hospital terciário. Todos os dados deste estudo referem-se à primeira visita de um ensaio clínico delineado para testar tanto a hipótese da associação da VOP com LOA e outras hipóteses a serem testadas no acompanhamento.

O tamanho da amostra para o ensaio clínico foi calculado usando a fórmula para comparação de dois grupos. Foram considerados um erro tipo I de 0,30, e uma proporção de não ocorrência de eventos cardiovasculares de 87,7% para o grupo controle e de 96,5% (com uma porcentagem a mais de 10% para não eventos) para o grupo experimental. Assim, uma amostra de 35 participantes foi obtida de cada grupo, mas decidimos expandir o tamanho amostral devido às possíveis perdas de seguimento.

Pacientes com hipertensão, usando ou não medicamentos anti-hipertensivos, foram recrutados como participantes.

### Critérios de inclusão

Pacientes com idade igual ou superior a 18 anos, hipertensos – pela aferição da pressão arterial (PA), que necessitavam de tratamento farmacológico^4^ foram incluídos no estudo.

### Critérios de exclusão

Os critérios de exclusão foram: participação em outros protocolos de pesquisa por menos de um ano, presença de doenças crônicas em estágios terminais à critério do investigador; doenças cardiovasculares anteriores (conhecida ou sintomática), incluindo doença arterial coronariana (infarto do miocárdio, angina, cirurgia prévia de *bypass* da artéria coronária, ou angioplastia), ou acidente vascular cerebral (ataque isquêmico transitório) durante os últimos seis meses. Os critérios de exclusão para doenças cardiovasculares prévias foram definidos usando informações coletadas dos pacientes (entrevistas diretas ou exames complementares).

### Coleta de dados e procedimentos do estudo

Os participantes foram entrevistados usando um questionário estruturado e medidas antropométricas (peso e altura). Além disso, dados de sexo, idade, e uso de medicamentos anti-hipertensivos foram coletados. O índice de massa corporal (IMC) foi calculado como peso (Kg) dividido pela altura (m) ao quadrado, e sobrepeso foi definido como IMC ≥ 25 kg/m^2^.^22^

As categorias de PA periférica foram definidas e medidas usando aparelhos automáticos OMRON®1100 seguindo-se as Diretrizes Brasileiras de Hipertensão Arterial – 2020.^4^ Foi considerada para análise a média de duas medidas de PA no mesmo braço, conduzidas em intervalos de dois minutos, foi considerada. Uma PA não controlada foi considerada como PA sistólica média ≥ 140 mmHg ou PA diastólica média ≥ 90 mmHg.

Parâmetros centrais foram avaliados de maneira não invasiva usando o método oscilométrico validado DynaMAPA AOP Cardios® (IEM, Stolber, Alemanha). Os procedimentos foram realizados pela mesma pessoa usando o mesmo equipamento usando o protocolo validado C1 PWV incluindo três medidas no mesmo braço em intervalos de um minuto.^23 - 26^ A pessoa responsável pelas medidas tem mais de cinco anos de experiência com métodos de medidas de pressão central.

A ultrassonografia com doppler das carótidas e a ecocardiografia foram realizadas seguindo-se as diretrizes dos consensos americano^27^e europeu.^28 , 29^ Esses foram conduzidos por um único observador, com mais de 10 anos de experiência, usando um sistema de ultrassom Philips Affiniti 70 e um transdutor linear com 12-4 MHz de frequência para Doppler da carótida e transdutor setorial com frequência 4-2MHz para ecocardiografia transtorácica.

### Análises de LOA

As análises de LOA incluíram medida da EIMC, presença de placas ateromatosas, e HVE. A EIMC foi definida como uma espessura maior ou igual a 0,9mm,^4^ e presença de placas carotídeas no território carotídeo.^4^ A HVE foi definida como razão massa ventricular/área da superfície corporal (g/m^2^)>115 (homens) e >95 (mulheres).^4^

### Aspectos éticos

O presente estudo seguiu a Resolução brasileira regulatória de pesquisas e testes em seres humanos número 466/12. Este estudo foi aprovado pelo Comitê de Ética em Pesquisa do Hospital das Clínicas da Universidade Federal de Goiás (CAAE:89488218.0.1001.5078). Todos os procedimentos foram realizados após obtenção de consentimento por escrito dos participantes.

### Análise estatística

A análise estatística foi realizada usando o programa Stata versão 14.0. Estatísticas descritivas foram calculadas usando frequências absolutas e relativas para variáveis qualitativas. Médias e desvios-padrões foram calculados para as variáveis quantitativas com distribuição normal, e medianas e intervalos interquartis foram calculados para aquelas com distribuição assimétrica.

Curvas características de operação do receptor (ROC) foram construídas para determinar o melhor ponto de corte para a VOP para definição da EIMC, presença de HVE, e de placas na carótida. Para a construção da curva ROC,^30^ valores de sensibilidade e de especificidade foram testados para as três variáveis que determinaram LOA (sim ou não). Para a construção da curva, foi escolhido o ponto de corte que apresentou a melhor combinação de sensibilidade e especificidade nas três variáveis simultaneamente. O ponto de corte da VOP foi obtido pela análise da curva ROC para identificar o melhor parâmetro para determinar os três desfechos.

A sensibilidade e a especificidade (ROC) foram analisadas para estimar o poder discriminatório das variáveis independentes e identificar o valor da VOP associada com HVE, EIMC, e presença de placas carotídeas. Na comparação dos valores de corte, a melhor combinação de sensibilidade e de especificidade foi verificada gerando-se um valor definido. Assim, a VOP foi categorizada como menor ou maior que o valor definido para comparações com diferentes variáveis da amostra usando os seguintes testes: qui-quadrado (variáveis qualitativas), teste t não pareado (variáveis quantitativas com distribuição normal), ou teste de Mann-Whitney (variáveis quantitativas com distribuição assimétrica). A significância estatística foi estabelecida em p<0,05.

## Resultados

A Tabela 1 apresenta as características dos 119 pacientes incluídos no estudo.

**Tabela 1:** – Características dos participantes

Variável	Média ± DP ou mediana (IIQ)
**Idade (anos)**	60,38 ±10,31
**Índice de Massa Corporal (Kg/m** ^**2**^ **)**	29,07 (26,67-32,89)
**Variável**	n (%)
**Sexo**	
Masculino	36 (30,25)
Feminino	83 (69,75)
**Sobrepeso**	
Sim	102 (80,95)
Não	24 (19,05)
**Parâmetros de pressão central (mmHg)**	
Pressão sistólica central	123 (114 - 133)
Pressão diastólica central	85 (78 - 94)
Pressão sistólica periférica	131 (121 - 142)
Pressão diastólica periférica	84 (77 - 93)
Pressão de pulso central	37 (30 - 44)
Índice de aumento	27 (15 - 25)
Resistência vascular periférica	1,28 (1,16 – 1,46)
Velocidade de onda de pulso	8,9 ± 1,7
**Espessura da íntima-média**	
Normal	45 (37,82)
Aumentada	74 (62,18)
EIMC (mm)	0,89 ± 0,40
**Placas carotídeas**	
Sim	68 (57,14)
Não	51 (42,86)
Índice massa ventricular esquerda (g/m^2^)	90 (71,3 – 113,6)
Pressão arterial não controlada	49 (41,18)

*DP: Desvio Padrão; IIQ: Intervalo Interquartil; EIMC: espessura íntima-média carotídea.*

**Figura 1:**
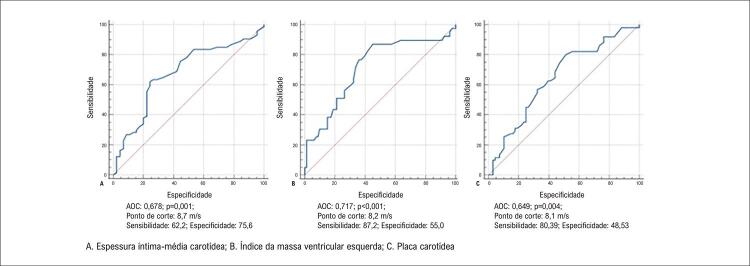
– Análise das curvas ROC.

Os pontos de corte para VOP indicando aumento da EIMC, presença de HVE, e presença de placas carotídeas definida usando a curva ROC foram 8,7 m/s, 8,2 m/s, e 8,1, respectivamente ( Figura 1 ).

Uma análise da sensibilidade e da especificidade de cada biomarcador foi realizada nos pontos de corte das três variáveis encontradas pela análise da curva ROC – IEMC (8,7), IMVE (8,2), e placas carotídeas (8,1). Na comparação dos pontos de corte, a melhor combinação de especificidade e sensibilidade foi observada para o valor 8,2 ( Tabela 2 ).

**Tabela 2:** – Sensibilidade e especificidade dos valores de velocidade de onda de pulso encontradas pela análise da curva ROC para espessura íntima-média carotídea (EIMC), hipertrofia ventricular esquerda (HVE), e presença de placas carotídeas

Ponto de corte	EIMC		HVE		Placas carotídeas	
	Sen. (%) (IC95%)	Esp. (%) (IC95%)	Sen. (%) (IC95%)	Esp. (%) (IC95%)	Sen. (%) (IC95%)	Esp. (%) (IC95%)
8,1	75,68 (64,3-8,9)	55,56 (40,0-70,4)	*-	-	80,39 (66,9-90,2)	48,53 (36,2-61,0)
8,2	68,92 (57,1-79,2)	57,78 (42,2-72,3)	87,18 (72,6-95,7)	55,00 (43,5-66,2)	74,51 (60,4-85,7)	52,94 (40,4-65,2)
8,7	62,16 (50,1-73,2)	75,56 (60,5-87,1)	74,36 (57,9-87,0)	65,00 (53,5-75,3)	60,78 (46,1-74,2)	61,76 (49,2-73,3)

EIMC: espessura íntima-média carotídea; HVE: hipertrofia ventricular esquerda (IMVE), Sen.: sensibilidade; Esp.: especificidade; *não houve pacientes com HVE na velocidade de 8,1m/s.

Um resumo é ilustrado na Figura Central .

A comparação de características sociodemográficas, variáveis clínicas, e valores hemodinâmicos e de pressão central entre indivíduos acima e abaixo do ponto de corte de 8,2 mostrou uma baixa frequência de sobrepeso e alta frequência de placas carotídeas em pacientes com ≥ 8,2 m/s. Ainda, esse grupo era mais velho e apresentaram valores mais altos de parâmetros hemodinâmicos centrais, HVE, e EIMC que indivíduos com VOP < 8,2 m/s ( Tabela 3 ).

**Tabela 3:** – Características da amostra e comparação de acordo com o valor da velocidade de onda de pulso (acima ou abaixo do ponto de corte de 8,2 m/s); n=119, 2018–2019

Variável	VOP < 8,2	VOP ≥ 8,2	p
**n**	49	70	
**Sexo**			0,090
Masculino	19 (38,78)	17 (24,29)	
Feminino	30 (61,22)	53 (75,71)	
**Sobrepeso**			0,010
Sim	45 (91,84)	51 (72,86)	
Não	4 (8,16)	19 (27,14)	
**IMC**			0,004
Normal	26 (57,78)	19 (42,22)	
Alterado	23 (31,08)	51 (68,92)	
**Placas carotídeas**			0,003
Não	36 (73,47)	32 (45,71)	
Sim	13 (26,53)	38 (54,29)	
**Idade (anos)**	51,59 ±5,85	66,54 ±8,04	0,001
**IMC (Kg/m** ^**2**^ **)**	29,33 (26,99–32,88)	28,45 (26,31–32,89)	0,205
PSC	117 (112–125)	126,5 (117–143)	<0,001
PDC	84 (76–92)	86 (78–96)	0,338
PSP	123 (119–131)	136 (125–149)	<0,001
PDP	83 (76–92)	85 (77–94)	0,595
PPC	31 (28–39)	40,5 (34–47)	<0,001
IA	16 (10–29)	30,5 (21–37)	<0,001
RVP	1,26 (1,16–1,43)	1,33 (1,2–1,46)	0,114
HVE	78,9 (68,1–93,6)	98 (79–123,8)	<0,001
EIMC	0,78 ± 0,41	0,96 +0,38	0,015

*PSC: pressão sistólica central; PDC: pressão diastólica central; PSP: pressão sistólica periférica; PPC: pressão de pulso central; IA: índice de aumento; RVP: resistência vascular periférica; VOP: velocidade de onda de pulso; IMC: índice de massa corporal; HVE: hipertrofia ventricular esquerda; EIMC: espessura íntima-média carotídea; PDP: pressão diastólica periférica.*

## Discussão

Neste estudo, uma VOP acima de 8,2m/s foi estatisticamente associada com EIMC aumentada, presença de placas carotídeas, e HVE. De acordo com a análise ROC, o ponto de corte mostrou melhor sensibilidade que outros valores de VOP.

Evidentemente, modelos de avaliação de risco capazes de identificar indivíduos com maior probabilidade de apresentar complicações em estágios iniciais da doença são desejáveis e necessários para reduzir risco residual. Uma meta-análise mostrou que o valor da VOP bem como um biomarcardor (LOA) apresenta importantes diferenças clínicas em indivíduos com risco moderado ou intermediário, demonstrando um aumento de 13% na classificação de risco global em 10 anos.^15^

Estudos definindo valores de referência da VOP em populações saudáveis e em populações em risco CV foram publicados no início da década de 20.^19 , 31 , 32^ Valores de VOP acima de 10m/s já foram estabelecidos como LOA nas principais diretrizes.^4 - 6^ Alguns estudos abordaram a distribuição de percentis; contudo, não há pesquisa sobre pontos de corte para normalidade e marcadores de risco CV.

Uma meta-análise identificou limiares de desempenho preditivo da VOP, os pontos de corte foram 10,7 m/s para mortalidade CV (AUC 0,75 [IC95%, 0,69-0,81]) e 11,5 m/s para mortalidade por todas as causas (AUC 0,78 [IC95%, 0,74-0,83]).^33^ Um estudo do tipo coorte encontrou que um ponto de corte para VOP > 9,4 m/s associou-se com uma maior incidência de mortalidade.^34^ Esses valores são maiores que aqueles encontrados no presente estudo. O estudo SPARTE usou um valor de VOP < 10m/s para direcionar o tratamento medicamentoso, mas ele não mostrou uma redução nos eventos CV maiores. Ainda, é questionável se os valores estratificados de VOP, e não o valor definido como LOA (10 m/s) foram usados para direcionar o tratamento, devido à diferença nos resultados. Além disso, o estudo SPARTE sofreu uma perda amostral devido à pandemia da COVID-19, o que pode ter afetado os resultados.^35^

Vários outros parâmetros para identificar lesões foram estudados. Um estudo chinês encontrou um ponto de corte ótimo da pressão arterial para identificar aterosclerose; os índices de pressão arterial tiveram um alto desempenho preditivo com um ponto de corte de 123,5/73,5 mmHg com um p<0,01.^36^ A VOP foi usada para avaliar escores de aterosclerose subclínica em indivíduos assintomáticos.^37^ A associação da VOP com EIMC, combinada com um índice de envelhecimento vascular ( *Vascular Aging Index, VAI* ) promove melhor predição de eventos CVs reclassificando pacientes sem eventos CVs prévios.^38^ Essa identificação precoce facilita uma abordagem individualizada.

Um estudo europeu distinguiu valores “normais” de valores “de referência” para VOP, no entanto, ainda não se sabe qual valor da VOP que apresenta a associação mais forte com biomarcadores. O presente estudo mostrou que uma VOP de 8,2m/s pode possibilitar uma identificação precoce de risco CV aumentado e ajuda estabelecer valores que podem ser considerados normais. A análise da VOP apresenta vantagens em relação aos testes diagnósticos, por exemplo, ela reduz demandas sobre o sistema de saúde e é altamente acessível, menos invasivo, mais seguro, mais barato, mais rápido e menos desconfortável fisicamente e psicologicamente para os pacientes.^39^

Este estudo não analisou um novo limiar para LOA, mas determinou um ponto de corte para VOP a partir de valores de referência previamente estabelecidos.^11 , 19^ O estudo definiu um valor que consegue identificar o desenvolvimento precoce de LOA e estabelecer valores de VOP que possam ser considerados anormais.

Uma associação significativa foi encontrada entre os biomarcadores e valores de VOP > 8,2m/s. Esses achados indicam que uma VOP menor que 10m/s, mas superior a 8,2 m/s deve ser considerada como ponto de corte associado com EIMC aumentada, presença de placas carotídeas, e HVE. Ainda, ela pode ajudar a estabelecer valores anormais em estudos populacionais de referência previamente publicados.

Este estudo tem algumas limitações. Uma das limitações foi o tamanho amostral, que pode ter sido responsável pelos valores de AUC, embora eles foram estatisticamente significativos.^40^ Estudos futuros incluindo uma amostra maior, vários centros, e maior duração podem fornecer valores de percentis de VOP relacionados à LOA. Ressaltamos que as medidas de PA foram aferidas apenas em um braço, mas como as diferenças entre os braços direito e esquerdo são raras, acreditamos que isso não relevante ao que nós encontramos. Os níveis de glicose e de colesterol, a presença ou não de diabetes e dados de raça/cor não estavam disponíveis de todos os pacientes e não foram considerados na análise do estudo.

Outros estudos são necessários para determinar o percentil que deve ser considerado na identificação do início de lesões subclínicas, e os valores que devem ser usados nos estudos de medidas de pressão central, descritos como valores de VOP normais e anormais.

## Conclusão

Encontrou-se uma associação significativa entre os biomarcadores e valores de VOP > 8,2m/s. Esses achados indicam que uma VOP acima de 8,2 m/s devem ser considerados como um ponto de corte associado com EIMC aumentada e a presença de placas carotídeas e HVE. O valor de 8,2 m/s pode ser mais sensível em identificar precocemente a existência de biomarcadores.
