# Supplementary material for: Velocidade de Onda de Pulso de 8,2m/s como Limiar Associado à Presença de Lesão de Órgão Alvo Cardiovascular
Source: Arq Bras Cardiol. 2023 Oct 26;120(10):e20220934. [Article in Portuguese] doi: 10.36660/abc.20220934 (PMC12080691; doi:10.36660/abc.20220934)
Supplement: Translation

# Translation: Pulse Wave Velocity of 8.2 m/s as a Threshold Associated with Cardiovascular Target Organ Damage Presence

**Authors:** Sayuri Inuzuka, Priscila Valverde de Oliveria Vitorino, Adriana Sebba Barroso, Fabrício Galdino Magalhães, Andrea Cristina Sousa, Robson Pierre Pacífico Alves, Victoria Alves Melo, Luiz Fernando de Oliveira, Ana Luiza Lima Sousa, Paulo Cesar B. Veiga Jardim, Antonio Coca, Weimar Kunz Sebba Barroso

**Affiliations:** 1 Brazil Universidade Federal de Goiás - Liga de Hipertensão Arterial, Goiânia, GO – Brazil; 2 Brazil Pontifícia Universidade Católica de Goiás - Escola de Ciências Sociais e da Saúde, Goiânia, GO – Brazil; 3 Spain Hypertension and Vascular Risk Unit. Hospital Clinic. University of Barcelona, Barcelona – Spain

**Keywords:** Vascular Stiffness, Pulse Wave Analysis, Arterial Pressure, Hypertension

## Abstract

**Background:**

Previous studies have established normal and reference values for Pulse Wave Velocity (PWV). However, the PWV value that has the strongest association with cardiovascular biomarkers remains poorly understood.

**Objective:**

This study aimed to determine the PWV value more likely to be associated with left ventricular hypertrophy (LVH), increased intima-media thickness (IMT), and presence of carotid plaques in patients with hypertension.

**Methods:**

This cross-sectional study included 119 patients. Analysis of receiver operating characteristic (ROC) curves was performed for each cardiovascular biomarker. Statistical significance was set at p < 0.05.

**Results:**

According to the ROC curve analysis, the PWV values were 8.1 m/s, 8.2 m/s, and 8.7 for the LVH, IMT, and presence of carotid plaques, respectively. A PWV value of 8.2 m/s was identified as the best parameter to determine the three TOD biomarkers. PWV above 8.2 m/s was associated with increased CIMT (p = 0.004) and the presence of carotid plaques (p = 0.003) and LVH (p<0.001). PWV above 8.2 showed greater sensitivity for increased CIMT (AUC = 0.678, sensitivity = 62.2), LVH (AUC = 0.717, sensitivity = 87.2), and the presence of plaques (AUC = 0.649, sensitivity = 74.51) in the ROC curve analysis.

**Conclusion:**

The PWV value 8.2 m/s was more sensitive in early identifying the existence of cardiovascular biomarkers of TOD.

## Introduction

A high number of patients with hypertension have subclinical lesions in the early stages of the disease that are not identified by traditional assessment models.^1 - 3^According to the main hypertension guidelines, more specific complementary tests for biomarker analysis are used for the early identification of cardiovascular (CV) damage.^4 - 6^

Carotid-femoral Pulse Wave Velocity (PWV) is the gold standard for arterial stiffness measurement because it is non-invasive, simple, accurate, reproducible, and has predictive value.^7 , 8^Stratified values of PWV are available for healthy individuals and those with increased CV risk. In addition, an association between PWV and target organ damage (TOD) has been established in patients with hypertension.^9 - 11^

Studies have shown that PWV is a predictor of CV events and mortality.^3 , 12 - 14^The use of PWV in addition to the traditional CV risk factors improves risk stratification.^15 , 16^A 10 m/s cut-off value for PWV was established as a definition of vascular TOD in previous studies and guidelines.^4 - 6^Additionally, PWV above 10 m/s has been associated with biomarkers of structural changes in the left ventricle chamber and carotid arteries, as well as with an increase in CV mortality.^16 - 18^

Arterial stiffness associated with age and sex and values stratified using the tonometric method have been established in previous studies, mostly conducted in Europe.^9 , 19 , 20^A European study^20^distinguished “normal” and “reference values.” While “normal” provides a physiological range, “reference” indicates the extent to which a population does not have noticeable CV diseases. Recently, reference values using the oscillometric method^10 , 11 , 21^were classified into with and without CV risk factors and stratified by age and sex.^11^

There is a gap in the existing literature in determining the ideal percentile of PWV to identify the normal value or the beginning of CV damage. The present study was aimed to identify the PWV value, not as a TOD, but with the strongest association with the following biomarkers: carotid intima-media thickness (CIMT), presence of atheromatous plaques, and left ventricular hypertrophy (LVH).

## Methods

This cross-sectional study was conducted between October 2018 and March 2019 in a multidisciplinary outpatient service of a tertiary hospital. All the data at this study concerns to the first visit of a clinical trial designed to test both this hypothesis of association of PWV with TOD and other hypotheses to be tested in the longitudinal follow-up.

The sample size for the clinical trial was calculated using the formula for comparing two groups. A type I error of 0.30, and a proportion of non-occurrence of cardiovascular events of 87.7% for the control group and 96.5% (with a plus percentage of 10% for non-events) for the experimental group were considered. Therefore, a sample of 35 participants was obtained for each group, but we have decided to expand the sample size due to possible loss of follow-up.

Patients with hypertension, taking or not taking antihypertensive drugs, were recruited as participants.

### Inclusion criteria

Patients aged 18 years or above, having hypertension as assessed through office blood pressure (BP) measurement, and requiring pharmacological treatment^4^were included in the study.

### Exclusion criteria

The exclusion criteria were participation in other research protocols for less than one year; presence of chronic diseases in the terminal stages at the investigator’s discretion; previous cardiovascular diseases (known or symptomatic), including coronary artery disease (myocardial infarction, angina, previous coronary artery bypass graft surgery, or angioplasty), or stroke (ischemic or transient ischemic attack) during the last six months. Exclusion criteria for previous cardiovascular diseases were defined using information collected from the patients (direct interviews or complementary examinations).

### Data collection and study procedures

The participants were interviewed using a structured questionnaire and anthropometric measurements (weight and height). Additionally, information on sex, age, and the use of antihypertensive medications was collected. Body mass index (BMI) was calculated as weight (kg) divided by height (m) squared, and overweight was defined as BMI ≥ 25 kg/m^2^.^22^

Peripheral BP categories were defined and measured using OMRON^®^1100 automatic devices according to the 2020 Brazilian Guidelines of Hypertension.^4^The average of the two BP measurements on the same arm, conducted at two-minute interval, was considered in this study. Uncontrolled BP was considered with mean systolic pressure ≥ 140 mmHg or diastolic pressure ≥ 90 mmHg.

Central parameters were assessed non-invasively using the validated DynaMAPA AOP Cardios® oscillometric method (IEM, Stolber, Germany). The procedures were performed by the same person and device using the C1 PWV-validated protocol including three measurements at same arm with 1-minute interval.^23 - 26^The person responsible for the measurements has more than five years of experience with central pressure measurement methodology.

Carotid doppler ultrasound and echocardiographic evaluations were performed following the American^27^and the European^28 , 29^consensus guidelines. These were conducted by a single observer, with more than 10 years of experience, using a Philips Affiniti 70 ultrasound system and a linear transducer with a 12-4 MHz frequency for carotid doppler and sectorial probe 4-2 MHz frequency for transthoracic echocardiography.

### Target organ damage analyses

TOD analyses included the assessment of CIMT, presence of atheromatous plaques, and LVH. CIMT was defined as a thickness greater than or equal to 0.9mm,^4^and presence of carotid plaques in carotid territory.^4^LVH was defined as left ventricular mass/body surface area (g/m^2^) >115 (men) and >95 (women).^4^

### Ethical aspects

The present study followed the Brazilian Ethics Regulatory Resolution No. 466/12. This study was approved by the Ethics Committee of the Hospital das Clínicas da Universidade Federal de Goiás (CAAE:89488218.0.1001.5078). All study procedures were performed after obtaining written informed consent from the participants.

### Statistical analysis

Statistical analysis was performed using Stata software version 14.0. Descriptive statistics were calculated using absolute and relative frequencies for qualitative variables. The Kolmogorov–Smirnov test was used to verify the data distribution of the variables.

Mean and standard deviation were calculated for quantitative variables with a normal distribution, while median and interquartile range were calculated for those with a skewed distribution.

Receiver operating characteristic (ROC) curves were constructed to determine the best PWV cut-off point for defining increased CIMT, presence of LVH, and presence of carotid plaques. For the construction of the ROC curve,^30^sensitivity and specificity values were tested for the three variables that determined TOD (yes or no). For the construction of the curve, the cut-off point that presented the best combination of sensitivity and specificity in the three variables simultaneously was chosen. The PWV cut-off point was obtained through the ROC analysis to identify the best parameter for determining the three outcomes.

Sensitivity and specificity (ROC) were analyzed to estimate the discriminating power of the independent variables to identify the PWV value associated with LVH, increased IMT, and the presence of carotid plaques. When comparing the cut-off values, the best combination of sensitivity and specificity was verified by generating a defined value. Accordingly, the PWV was categorized as less than or greater than the defined value for comparisons with several sample variables established using the following tests: chi-square (qualitative variables), unpaired t-test (quantitative variables with normal distribution), or Mann–Whitney U test (quantitative variables with skewed distribution). Statistical significance was set at p <0.05.

## Results

Table 1 presents the characteristics of the 119 patients included in this study.

**Table 1:** – Characteristics of the participants

Variable	Average ± SD or Median (IQR)
**Age (years)**	60.38 ±10.31
**Body mass index (Kg/m2)**	29.07 (26.67-32.89)
**Variable**	n (%)
**Sex**	
Men	36 (30.25)
Women	83 (69.75)
**Overweight**	
Yes	102 (80.95)
No	24 (19.05)
**Central blood pressure parameters (mmHg)**	
Central SBP	123 (114 - 133)
Central DBP	85 (78 - 94)
Peripheral SBP	131 (121 - 142)
Peripheral DBP	84 (77 - 93)
Central pulse pressure	37 (30 - 44)
Augmentation index	27 (15 - 25)
Peripheral vascular resistance	1.28 (1.16 – 1.46)
Pulse wave velocity (m/s)	8.9 ± 1.7
**CIMT**	
Normal	45 (37.82)
Thickened	74 (62.18)
CIMT (mm)	0.89 ± 0.40
**Carotid plaques**	
Yes	68 (57.14)
No	51 (42,86)
Left ventricular mass index (g/m^2^)	90 (71.3 – 113.6)
Uncontrolled blood pressure	49 (41.18)

*DBP: diastolic blood pressure; SBP: systolic blood pressure; SD: standard deviation; IQR: interquartile range; CIMT: carotid intima-media thickness (CIMT).*

The cut-off points for the PWV indicating increased CIMT, presence of LVH, and presence of carotid plaques defined using the ROC were 8.7 m/s, 8.2 m/s, and 8.1, respectively ( Figure 1 ).

**Figure 1:** – Analysis of ROC curves.

An analysis of the sensitivity and specificity of each biomarker was performed at the cut-off points of the three variables that were found through the ROC curve analysis – CIMT (8.7), LVMI (8.2), and carotid plaques (8.1). When comparing the cut-off points, the best combination of sensitivity and specificity was verified for the 8.2 value ( Table 2 ).

**Table 2:** – Sensitivity and specificity of pulse wave velocity values found through the ROC analysis for carotid intima-media thickness (CIMTC), left ventricular hypertrophy (LVH), and presence of carotid plaques

Cut-off	IMT		LVH		Carotid plaques	
	Sen. (%) (95% CI)	Spe. (%) (95% CI)	Sen. (%) (95% CI)	Spe. (%) (95% CI)	Sen. (%) (95% CI)	Spe. (%) (95% CI)
8.1	75.68 (64.3-8.9)	55.56 (40.0-70.4)	*-	-	80.39 (66.9-90.2)	48.53 (36.2-61.0)
8.2	68.92 (57.1-79.2)	57.78 (42.2-72.3)	87.18 (72.6-95.7)	55.00 (43.5-66.2)	74.51 (60.4-85.7)	52.94 (40.4-65.2)
8.7	62.16 (50.1-73.2)	75.56 (60.5-87.1)	74.36 (57.9-87.0)	65.00 (53.5-75.3)	60.78 (46.1-74.2)	61.76 (49.2-73.3)

CIMT: carotid intima-media thickness; Sen.: sensitivity; Spe.: specificity. * There was no participant with LVH at a speed of 8.1m/s

A summary is found in the Central Figure .

The comparison of sociodemographic characteristics, clinical variables, pressure, and central hemodynamic values between individuals above or below the cut-off point of 8.2 showed a low frequency of overweight and high frequency of carotid plaques among patients with PWV > 8.2 m/s. Additionally, this group of were older and had higher central hemodynamic parameters, LVH, and CIMT than individuals with < 8.2 m/s PWV value ( Table 3 ).

**Table 3:** – Sample characteristics and comparison according to the PWV value above or below the cut-off value of 8.2 m/s, n=119, 2018–2019

Variable	PWV < 8.2	PWV ≥ 8.2	p
**n**	49	70	
**Sex**			0.090
Male	19 (38.78)	17 (24.29)	
Female	30 (61.22)	53 (75.71)	
**Overweight**			0.010
Yes	45 (91.84)	51 (72.86)	
No	4 (8.16)	19 (27.14)	
**BMI**			0.004
Normal	26 (57.78)	19 (42.22)	
Altered	23 (31.08)	51 (68.92)	
**Carotid plaques**			0.003
No	36 (73.47)	32 (45.71)	
Yes	13 (26.53)	38 (54.29)	
**Age (years)**	51.59 ±5.85	66.54 ±8.04	0.001
**BMI (Kg/m** ^**2**^ **)**	29.33 (26.99–32.88)	28.45 (26.31–32.89)	0.205
cSBP	117 (112–125)	126.5 (117–143)	<0.001
cDBP	84 (76–92)	86 (78–96)	0.338
pSBP	123 (119–131)	136 (125–149)	<0.001
pDBP	83 (76–92)	85 (77–94)	0.595
cPP	31 (28–39)	40,5 (34–47)	<0.001
AIX	16 (10–29)	30.5 (21–37)	<0.001
PVR	1.26 (1.16–1.43)	1.33 (1.2–1.46)	0.114
LVH	78.9 (68.1–93.6)	98 (79–123.8)	<0.001
IMT	0.78 ± 0.41	0.96 +0.38	0.015

*AIX: augmentation index; BMI: body mass index; cDBP: central diastolic blood pressure; cPP: central pulse pressure; cSBP: central systolic blood pressure; IMT: intima-media thickness; LVH: left ventricular hypertrophy; pDBP: peripheral diastolic blood pressure; pSBP: peripheral systolic blood pressure; PVR: peripheral vascular resistance; PWV: pulse wave velocity.*

## Discussion

In this study, a PWV above 8.2 m/s was statistically associated with increased CIMT, presence of carotid plaques, and LVH. According to the ROC analysis, the cut-off point showed better sensitivity than the other PWV values.

Evidently, risk assessment models capable of identifying individuals who are most likely to have complications in the early stages of the disease are desirable and required to reduce residual risk. A meta-analysis showed that the PWV value as a biomarker (TOD) presents important clinical differences in individuals with moderate or intermediate risk, demonstrating a 13% increase in the global risk classification within 10 years.^15^

Studies defining PWV reference values in healthy and CV risk populations have been published since the early 2000’s.^19 , 31 , 32^PWV values above 10 m/s have already been established by the main guidelines as TOD.^4 - 6^Some studies have addressed the distribution of percentiles, however, there is a lack of research concerning cut-off points for normality and CV risk markers.

A meta-analysis identified thresholds for predictive performance of PWV, the cut-off points were 10.7 m/s for CV mortality (AUC 0.75 [95% CI, 0.69–0.81]) and 11.5 m/s for all-cause mortality (AUC 0.78 [95% CI, 0.74–0.83]).^33^A cohort study found a PWV cut-off point > 9.4 m/s to be associated with a higher incidence of mortality.^34^These values are higher than those found in the present study. The SPARTE study used a value of PWV < 10 m/s to guide drug treatment; however, it did not show a reduction in major CV events.^35^Also, it is questionable whether the stratified PWV values, rather than the value defined as TOD (10 m/s) were used to guide the treatment, due to the difference in results. Furthermore, the SPARTE study had a sample loss due to the COVID-19 pandemic, which may have affected their results.

Various other parameters have been studied to identify lesions. A Chinese study found an optimal blood pressure cut-off point to identify atherosclerosis; the blood pressure indexes had a high predictive performance with an optimal cut-off point of 123.5/73.5 mmHg at p<0.01.^36^The PWV has been used to assess subclinical atherosclerosis scores in asymptomatic individuals.^37^The association of PWV with CIMT, combined with a vascular aging index (Vascular Aging Index, VAI) promotes better prediction of CV events by reclassifying patients with no previous CV events.^38^This early identification facilitates an individualized approach.

A European study^19^distinguished “normal” and “reference values” for PWV; however, the PWV value that has the greatest association with biomarkers remains poorly understood. The current study found that a PWV of 8.2 m/s may enable early identification of increased cardiovascular risk and help establish values that can be considered normal. The PWV analysis has advantages over diagnostic tests, such as it reduces demands on the healthcare system and is highly accessible, less invasive, less dangerous, less expensive, less time-consuming, and less physically and psychologically uncomfortable for patients.^39^

This study did not analyze a new threshold for TOD, but determined a cut-off point for PWV from the previously established reference values.^11 , 19^The study defined a value that can identify early TOD development and establish values of PWV that may be considered abnormal.

A significant association was found between the biomarkers and PWV values > 8.2 m/s. These findings indicate that a PWV lower than 10 m/s but higher than 8.2 m/s should be considered as the cut-off point associated with increased CIMT, presence of carotid plaques, and LVH. Furthermore, it may help establish values that can be considered abnormal in the previously published reference population studies.

This study had some limitations. One of the limitations was the sample size, which may have been responsible for the AUC values, although they were statistically significant.^40^Future studies with a large sample size, multiple centers, and longer study duration may provide percentile values of PWV related to TOD.

We point out that the BP measurement was performed in only one arm, but since the differences between the right and left arms are rare, we believe that it was not relevant to what we found. Glucose and cholesterol levels, the presence or not of diabetes and race/color data were not available for all patients and were not considered for the analysis of this study.

Further studies are required to determine the percentile that should be considered to identify the onset of subclinical lesions, and the values that should be used in reports of central pressure measurements, described as normal and abnormal PWV values.

## Conclusion

A significant association was found between the biomarkers and PWV values > 8.2 m/s. These findings indicate that a PWV above 8.2 m/s should be considered as a cut-off point associated with increased CIMT and the presence of carotid plaques and LVH. The 8.2 m/s value may be more sensitive in early identifying the existence of biomarkers.
